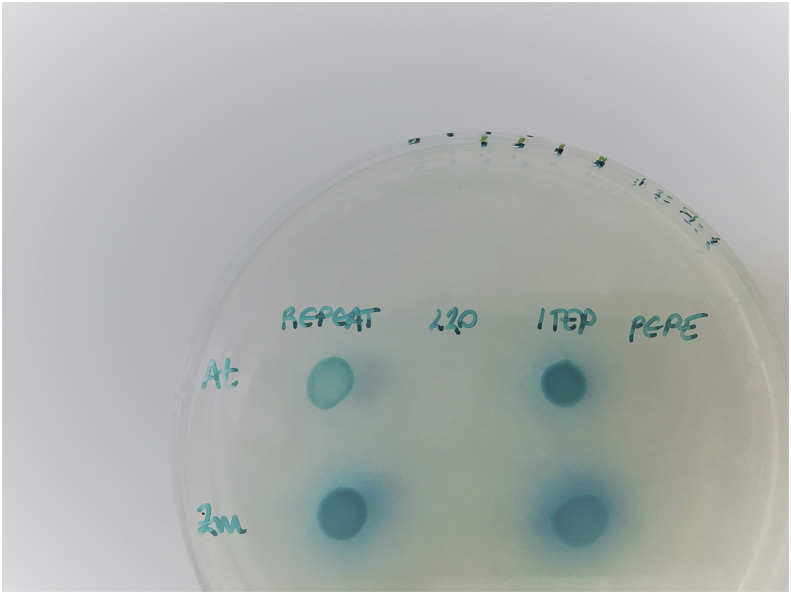# Qualitative and quantitative dataset of TROL protein interaction with C3 and C4 ferredoxin: NADP^+^ oxidoreductases

**DOI:** 10.1016/j.dib.2019.105038

**Published:** 2019-12-20

**Authors:** Anja Rac, Hrvoje Fulgosi

**Affiliations:** Laboratory for Molecular Plant Biology and Biotechnology, Division of Molecular Biology, Institute Ruđer Bošković, 10000 Zagreb, Croatia

**Keywords:** Photosynthesis, FNR, TROL, Protein interaction, Electron transport chain

## Abstract

Last step of electron transport from ferredoxin to NADP+ in photosynthesis light reactions catalyses ferredoxin: NADP^+^ oxidoreductase (FNR). FNR is present as soluble protein in stroma, but also bound to the protein complexes on the membrane with thylakoid rhodanase-like protein (TROL) and translocon on the inner envelope chloroplast membrane (Tic62), which have identical C terminal FNR binding domain [1,2]. During the electron transport, FNR anchored by TROL protein transfers electrons on NADP+ and forms NADPH which is then used in Calvin cycle as reducing agent. TROL is an integral membrane protein [3] with an inactive rhodanase-like domain (RHO) facing stroma which, as proposed earlier [4], could bind a small ligand leading to releasing or binding of FNR. FNR-TROL protein complex is necessary for optimal photosynthetic electron flow [1]. It has been shown that C4 plant maize FNR isomers have different N-terminal structure which determines binding affinity to protein complexes and different ratios of bound and unbound FNR in bundle sheath and mesophyll cells, depending on preferable photosynthetic electron transport [5]. Mutant Arabidopsis plant that contain maize FNR1 protein showed influence on dynamic association of FNR and change in excitation balance between photosystems which then influenced photo induced electron transport and finally photosynthesis [5]. In order to determine the influence of maize FNR1 on photosynthesis in C3 plants and difference in interaction strength with TROL, we preformed Yeast two hybrid screening, x-alpha-gal assay and β-galactosidase assay.

**Table:** Specifications Table

Subject	Biology, Biochemistry
Specific subject area	Protein interactions
Type of data	Image Chart
How data were acquired	Yeast two hybrid, β-galactosidase assay, X-alpha-gal assay, yeast transformation
Data format	Raw Analyzed
Parameters for data collection	Bait vector pBDGAL4 Cam and prey vectors pGADT7 and pADGAL4 were introduced in yeast strains AH109 and Y187. Bait polypeptides are C-terminal regions of protein TROL (ITEP) and protein Tic62 (IA2), protein TROL (220) and protein TROL (PEPEa). *Pisum sativum* (C3) FNR1 and maize (C4) FNR1 were introduced in prey vectors.
Description of data collection	After yeast transformation, selective medium without Leucine and Triptophan was used for transformants selection. For interaction screening we used selective plates lacking Leucine, Triptophan, Adenine and Histidine. β-galactosidase assay and X-alpha-gal assays were preformed according to manufacturer's instructions.
Data source location	Zagreb, Croatia
Data accessibility	Data is supplied in this article

**Table:** 

**Value of the Data** • Data assess qualitative and quantitative protein interaction of maize FNR1 in C3 plant • Interactions were tested with Yeast two hybrid screening, X-alpha-gal and β-galactosidase assay • This dataset gives insight in protein interactions for further experiments on FNR, TROL and photosynthesis especially for mutant C3 plants with C4 elements

## 1 Data

**Fig. 1:**
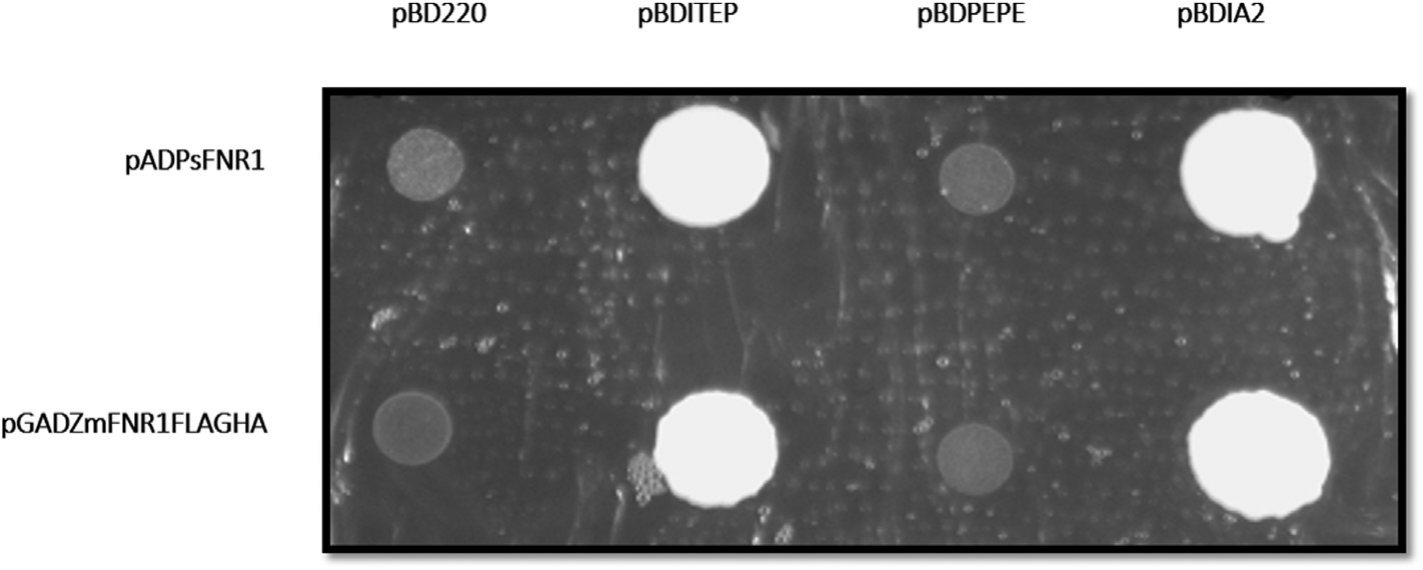
Yeast two hybrid protein interaction screening. Growth of yeast cells in different bait-prey combination. Upper and lower rows correspond to *Pisum sativum* and *Zea mays*, respectively.

**Fig. 2:**
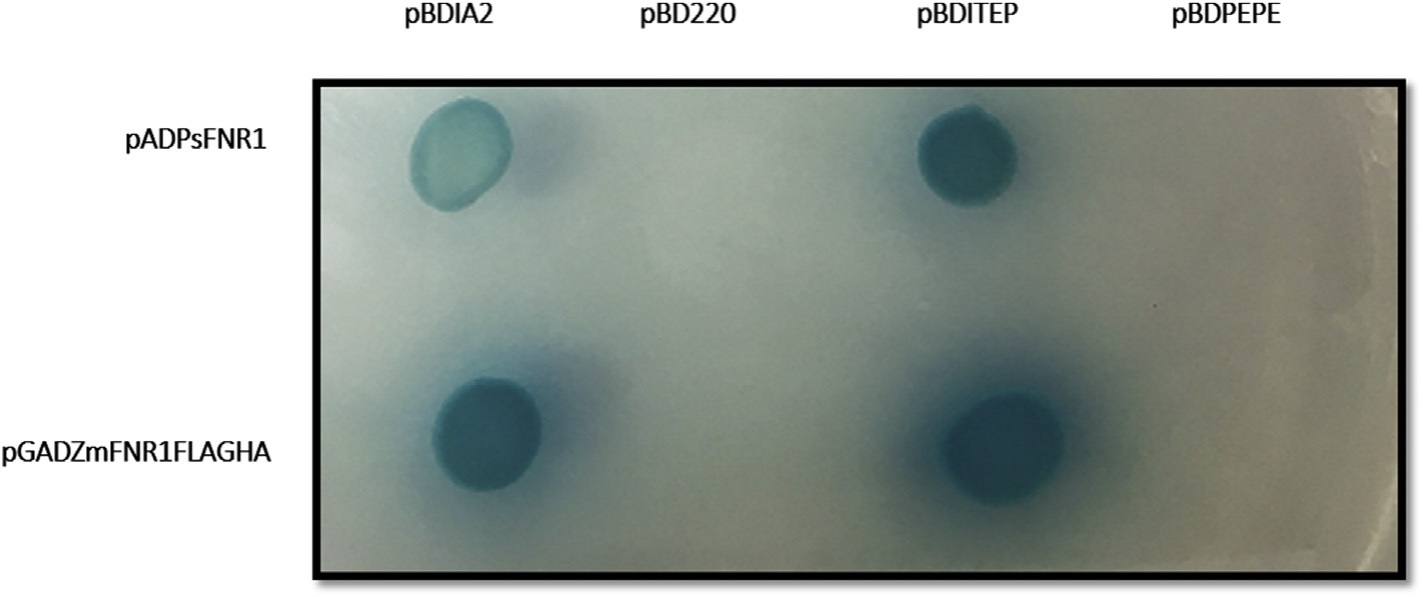
Semiquantitative yeast two hybrid protein interaction screening. Growth of yeast cells in different bait-prey combinations in the presence of X-alpha-gal. Upper and lower rows correspond to *Pisum sativum* and *Zea mays*, respectively.

The data indicates stronger interaction [4] between maize FNR1 with ITEP region of TROL [5] than one between pea FNR1 and ITEP. Yeast two hybrid interaction screening showed positive interactions between both FNR's with ITEP and IA2 region and negative with module 220 and PEPE (Fig. 1). Vigorous yeast cell growth and intensive blue color development from yeast MEL1 gene activation in X-alpha-gal assay was detected in ZmFNR1FLAGHA-ITEP protein interaction and lighter blue color was developed in interaction PsFNR1 with ITEP and IA2 regions and ZmFNR1FLAGHA with IA2 (Fig. 2). β-galactosidase assay showed that ZmFNR1FLAGHA-ITEP interaction is 10 times stronger than PsFNR1-ITEP interaction (Table 1). Raw data of β-galactosidase assay, Yeast two hybrid screening and X-alpha-gal assay figures can be found in Supplementary materials.

**Table 1:**
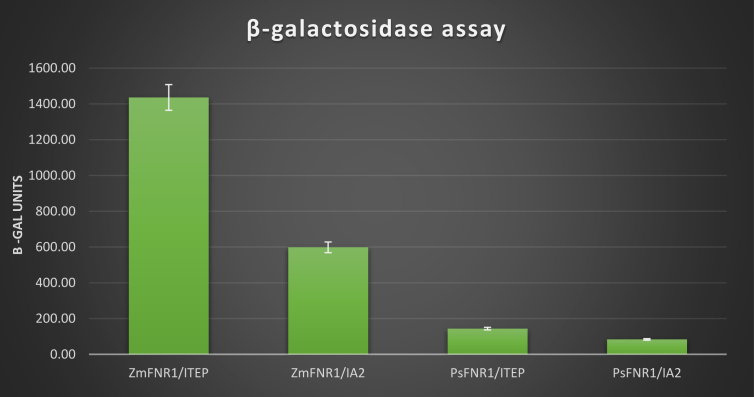
Yeast two hybrid bait-prey interaction quantified by β-galactosidase assay. Mean values with percentage error.

## 2 Experimental design, materials, and methods

C-terminal regions of TROL named ITEP (residues I429–P466) and Tic62 named IA2 (residues M263-S444), were introduced in bait vector pBD-Gal4 Cam, as well as additional regions of TROL which don't participate in TROL-FNR interaction - module 220 (residues N180–V220) and PEPE (residues A379-A419) [1,3]. IA2 C-terminal region was used as positive control [2] and empty bait vector as negative control. Prey proteins *Zea mays* ZmFNR1FLAGHA and *Pisum sativum* FNR1 (PsFNR1) were introduced in prey vectors pGADT7 and pAD-GAL4-2.1, respectively. After successful cloning, which was confirmed by sequencing, prey and bait vectors were introduced in AH109 (MATa, trp 1–901, leu2-3, 112, ura 3–52, his3-200, gal4△, gal80△, LYS2: GAL1_UAS_-GAL1_TATA_-HIS3, GAL2_UAS_-GAL2_TATA_-ADE2, URA3: MEL1_UAS_-MEL1_TATA_-LacZ) yeast cell line for Yeast two hybrid assay and X-alpha-galactosidase assay and for β-galactosidase assay in yeast cell line Y187 (MATα, ura3-52, his3-200, ade2-101, trp1-901, leu2-3, 112, gal4Δ, met–, gal80Δ, MEL1, URA3: GAL1UAS -GAL1TATA-lacZ).

Yeast cells were transformed by modified Li–O-Ac method. Cells were grown overnight in YPD medium (Yeast extract 10 g/l (w/v), Peptone 20 g/l (w/v), Glucose 20 g/l (w/v)) on 30 °C with constant mixing on rotator. One ml of cells was pelleted in a microfuge for 5 sec. prior to each transformation. Pellet was resuspended by shaking in 50–100 μl of the remaining medium. Two μl of carrier DNA (10 mg/ml, denatured by boiling) were then added, followed by addition of 1 μg of plasmid construct. Mixture was vortexed and 500 μl of PLATE mixture (90 ml 45% PEG, 10 ml 1 M Li–O-Ac, 1 ml 1 M Tris-Cl and 200 μl 0,5 M EDTA) was added. Fifty μl of 1 M DTT was added and the mixture was vortexed again, followed by 6 to 8-h incubation on room temperature. Cells were heat shocked for 10 minutes at 42 °C and plated on selective medium plates.

Transformants were selected on selective medium plates lacking Leucine and Tryptophan, grown for three days on 30 °C and transferred in same liquid medium overnight, 200 rpm on 30 °C. For protein-protein interaction screening cells were plated on selective medium lacking Leucine, Tryptophan, Histidine and Adenine and grown on 30 °C for 3 days. β-galactosidase assay is done according to manufacturer's instructions (Thermo Fischer), same as X-alpha-galactosidase assay (Takara).

## Figures and Tables

**Fig. S1:**
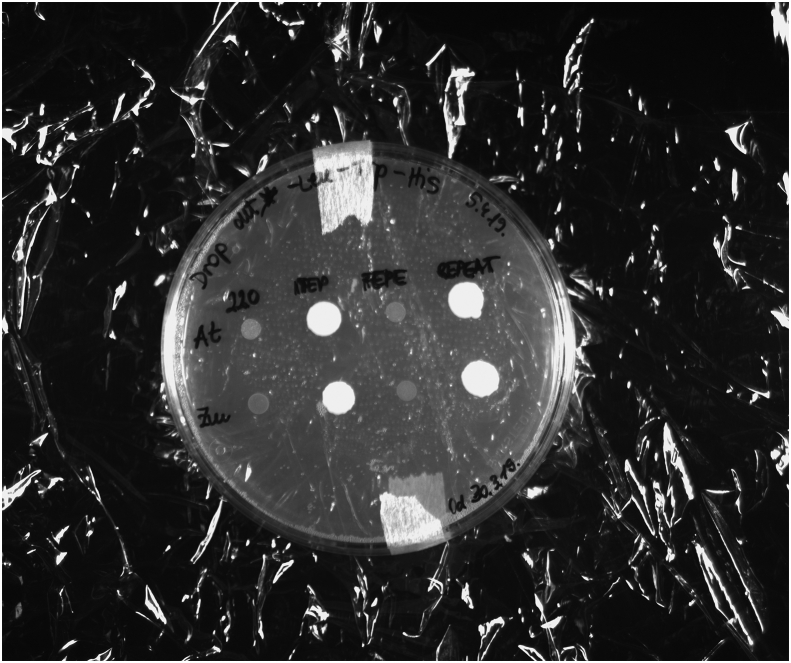


**Fig. S2:**